# Transcriptomics Insights into Targeting CK2 Complex in *Cryptococcus neoformans*: Implications for Large-Scale Antifungal Virtual Screening

**DOI:** 10.22034/cmm.2024.345250.1548

**Published:** 2024-12-09

**Authors:** Fadia Falah Hassan, Mohammed Hussein Mushrif, Mohammed F Hamdi, Ahmed AbdulJabbar Suleiman

**Affiliations:** 1 Biology department, College of education for Pure Science-Ibn Alhaitham, University of Baghdad, Baghdad, Iraq; 2 Medical Microbiology department, College of Medicine, Al-Iraqia University, Baghdad, Iraq; 3 Department of Medical Laboratories Techniques, Al maarif University College, Al Anbar, 31001, Iraq; 4 Department of Biotechnology, College of Science, University of Anbar, Anbar, 31001, Iraq

**Keywords:** *Cryptococcus neoformans*, Ck2 Complex, pathogenic proteins, FDA-approved drugs, therapeutic targets

## Abstract

**Background and purpose:**

*Cryptococcus neoformans* is a pathogenic fungus that causes fungal meningitis and other infections in immunocompromised patients. The casein kinase 2 (Ck2) complex regulates cellular processes. This study provides transcriptomics and functional insights into the Ck2 complex and other pathogenic proteins of *Cryptococcus neoformans* as therapeutic targets.

**Materials and Methods:**

The study used computational methods to explore the transcriptomic and functional aspects of the Ck2 complex and other pathogenic proteins in *Cryptococcus neoformans*. RNA-sequencing analysis of control and experimental cell cultures under three different conditions (cka1Δ mutant vs wild, ckaΔ, ckb1Δ, ckb2Δ [triple] mutants vs wild, and wild vs all mutants) was performed, followed by the STRING analysis of the dysregulated genes to identify the protein-protein interactions, while Cytoscape was used to identify the hub genes in all three conditions.

**Results:**

The RNA-sequencing analysis resulted in various dysregulated genes such as 936 (cka1Δ mutant vs wild), 1154 (triple vs wild), and 1159 (wild vs all mutants). Cellular components, molecular functions, and KEGG pathways in three conditions. The hub genes that elevated the most, Q5KFT2_CRYNJ, ARO1_CRYNJ, Q5KL19_CRYNJ, Q5KC42_CRYNJ, Q5KNI6_CRYNJ, Q5KCS1_CRYNJ, Q5KNH2_CRYNJ, Q5KA46_CRYNJ, Q5KEV1_CRYNJ, Q5KFT0_CRYNJ, Q5KAB9_CRYNJ, Q5KN73_CRYNJ, Q5KLJ6_CRYNJ, and Q5KHQ2_CRYNJ, were selected for FDA-approved drugs screening using GNINA, resulting in three potential drugs (amphotericin B, idarubicin, and candicidin) for respective proteins.

**Conclusions:**

The Ck2 complex in *C. neoformans* regulates cellular processes, including proliferation and apoptosis. Disruption of this complex affects cellular functions. This study identifies deletion mutations and pathogenic proteins, revealing top-performing drugs. Further clinical investigations are needed to confirm these findings.

## Introduction

*Cryptococcus neoformans*, a pathogenic fungus, is the primary cause of fungal meningitis and infections, causing malaise and mortality in humans and animals. [ 2 ]. This fungus is a potent pathogen that primarily affects patients with compromised immune function due to serious diseases like HIV/AIDS, cancer, steroid therapy, and chemotherapy. [ 3 ]. Several comprehensive studies have been performed owing to its biomedical importance and genetic influence [ 4 ].

From a statistical perspective, due to cryptococcal meningitis, over 600,000 fatalities, along with approximately millions of new life-threatening cases, were reported [ 1 , 5 ]. According to the CDC report, in 2022, each year, over 152,000 instances, as well as 112,000 deaths related to cryptococcal meningitis among HIV/AIDS patients, were recorded globally [ 6 ]. In 2014, an annual incidence of 223,100 cases around the globe followed by 180,100 deaths, in which Sub-Saharan Africa showed greater susceptibility towards the cryptococcal disease [ 7 , 8 ]. In the USA, the CDC reports the prevalence of about one million new cryptococcal meningitis cases yearly across the globe. Therefore, the infections caused by C. neoformans have become an indispensable health concern worldwide [ 9 ].

Cryptococcal meningitis is linked to various immune system-compromised conditions like AIDS, diabetes, cancer, autoimmune diseases, and lymphoproliferative malignancy. Liver cirrhosis is also a risk factor, and several factors are linked to T-cell dysfunction [ 10 , 11 , 12 ]. 

 This disease is transmitted through inhalation via the airways, and the lungs are considered the primary gateway [ 13 ]. The C. neoformans can move into the blood-brain barrier and bloodstream and cause several central nervous system (CNS) disorders as well as meningitis in case it is not carried in the lungs [ 2 ]. Cryptococcus neoformans, an environmental pathogen, causes cryptococcal meningitis, causing symptoms like fever, nausea, vomiting, headache, neck pain, and light sensitivity. Infections with the lungs cause pneumonia-like illness, including shortness of breath, chest pain, cough, and fever [ 10 , 14 ].

 The WHO recommends three steps for treatments against cryptococcal meningitis: induction, consolidation, and maintenance. Induction involves high-dose drugs, consolidation involves monotherapy, and maintenance uses low-dose drugs until the immune system recovers. Various antifungal drugs have been used so far [ 15 , 16 , 17 ]. 

 Protein kinase 2, also known as Casein kinase 2, is an active serine/threonine complex involved in regulating cell processes like proliferation, signaling pathways, growth, angiogenesis, and apoptosis, and its disruption can impact cellular functions[ 1 , 17 - 19 ].

 Four major signaling pathways are crucial for C. neoformans' virulence: calcineurin, MAPK, cAMP, and TOR. These pathways respond to calcium homeostasis, elongate hyphae, and regulate energy levels and cellular nutrients. PKCA1 signal transduction regulates cell wall biogenesis and integrity [ 20 ].

 The study aimed to identify potential targets for the Ck2 complex, including knock-out substrates cka1, ckb1, and ckb2, and pathogenic proteins, using computational methods like RNA-sequencing, functional enrichment, and virtual screening.

## Materials and methods

### RNA-sequencing data retrieval, read quantification, and differential gene expression analysis

 The NCBI gene expression omnibus (GEO), a repository with high throughput gene expression and functional genomics data [ 21 ], was used to download feature count data from experimental and control cell cultures of *C. neoformans*. Paired-end data sequenced using Illumina HiSeq 4000 platform was used comprising 9 samples; 3 samples (wild) were from controlled cultures, and the remaining 6 samples (ckb1Δ, ckb2Δ, and cka1Δ experimental) were from the experimental cultures.

Differential gene expression was performed using DESeq2 2.30.0 package in R 4.2.2 between control and experimental samples, identifying significant genes based on p-value < 0.05 and 1 < LogFC < -1, revealing up and down-regulated genes.

### Protein-protein interaction, hub genes identification, and functional enrichment analysis

The search tool for retrieval of interacting genes/proteins (STRING, version: 12.0) database (https://string-db.org/; December 11) was utilized to analyze the protein-protein interactions (PPI) by inputting the target genes. STRING is an organized database that assimilates and curates PPIs by utilizing automated text-mining techniques [ 22 ]. Furthermore, hub genes were identified using Cytoscape software (version 3.10.1) [ 23 ].

The identified dysregulated genes acquired from RNA-sequencing were utilized for performing the gene ontologies (GO) and pathway enrichment analysis via a web-based tool, YeastEnrichr (https://maayanlab.cloud/YeastEnrichr/; December 12) [ 24 ].

### Structure retrieval of selected target proteins and domain analysis

The 3D protein structures were retrieved by utilizing the Python package OptimalPDB (https://github.com/waqarhanif-biocode/optimalpdb; December 14), which retrieves PDB database structures, a worldwide repository containing the macromolecules verified 3D structures along with their complexes [ 25 ]. Moreover, the AlphaFold database (https://alphafold.ebi.ac.uk/; December 14) [ 26 ] was used to retrieve the structures (PDB) with unmodelled regions < 70%.

 Furthermore, the InterPro public database (https://www.ebi.ac.uk/interpro/), which includes the protein functional sites, domains, and families [ 27 ], was employed for interpreting the functional domain of all the selected proteins.

### Natural compounds retrieval

 A literature review was conducted to identify novel therapeutic agents for cryptococcal meningitis. ChEMBL database was utilized to retrieve 2,042 compounds in SMILES notation , which were converted into. *pdb* format for further analysis, aiming to find antifungal, FDA-approved, and bioactive compounds for treating *C. neoformans*. [ 28 , 29 ]. 

### Selected protein targets virtual screening against natural compounds

 GNINA, a deep learning framework for molecular docking, was used to screen the selected protein targets against the selected natural compounds (https://github.com/gnina/gnina; December 18). It resulted in 9 poses for each ligand with its respective protein, and the best affinity pose was selected for interaction analysis, which was conducted using the Protein-ligand interaction profiler (PLIP) web-based tool, which showed the protein residues and ligand interactions.

## Results

### Identification of differentially expressed genes

The differentially expressed genes resulted in 673 upregulated genes in the cka1Δ vs wild-type strain condition, while 263 genes displayed downregulation. In the triple vs wild-type strain condition, 791 genes showed upregulation and 363 genes showed downregulation. In the wild-type strain vs all mutants condition, 238 genes were upregulated and 921 downregulated, as illustrated in Supplementary Document 1 Figure S1 (A-C).

### GO terms for dysregulated genes

The upregulated genes in BP of cka1Δ vs wild-type strain exhibited the enrichment in the glycerophospholipid catabolic and metabolic processes, glycerolipid and phospholipid catabolic processes, cellular response to decreased oxygen, pH, and alkaline pH levels, fungal cell wall biogenesis. However, the downregulated genes showed enhancement in the histone and histone-lysine methylation, L-methionine salvage from methylthioadenosine (Supplementary Document 1 Figure S2 and S11). Moreover, the MF disclosed that the upregulated genes play a pivotal role in the lysophospholipase activity, phospholipase and phospholipase A2 activity, DNA binding, sequence-specific DNA binding, carboxylic ester hydrolase activity, transcriptional repressor, and factor activities whereas the downregulated genes were involved in four-way junction helicase activity, single-stranded DNA dependent ATP-dependent DNA helicase activity. (Supplementary Document 1 Figure S4 and S13). Additionally, these upregulated genes in the CC manifested their role in the nucleus, whereas in downregulated genes, these genes exhibited enrichment in the nuclear nucleosome and chromatin (Supplementary Document 1 Figures S3 and S12).

 The BP revealed that upregulated genes are involved in phosphatidylcholine metabolic process, mRNA cleavage, and cellular response to decreased oxygen levels, while downregulated genes positively regulate DNA-templated transcription, initiation, and L-methionine salvage from methylthioadenosine (Supplementary Document 1 Figures S5 and S14). Furthermore, the triple conditions’ upregulated genes in the MF were enriched in the lysophospholipase activity, RNA helicase, and ATP-dependent RNA helicase activity (Supplementary Document 1 Figures S7 and S16). The CC revealed that upregulated genes were involved in the ERMES complex, phagophore, and U6 snRNP, while downregulated genes were involved in chromatin, alpha DNA polymerase primase complex, and nuclear nucleosome (Supplementary Document 1 Figure S6 and S15). 

 The BP of upregulated genes in the wild-type strain vs all mutant conditions appear to be enriched in histone and histone-lysine methylation, L-methionine salvage from methylthioadenosine. In contrast, the downregulated genes show the interruption in cellular response to decreased oxygen level, and regulation of mRNA poly(A) tail shortening (Supplementary Document 1 Figure S8 and S17). The upregulated genes MF exhibited their roles in the four-way junction activity, single-stranded DNA-dependent ATP-dependent DNA helicase activity. Conversely, the downregulated genes were involved in the polypyrimidine tract binding and lysophospholipase activity (Supplementary Document 1 Figure S10 and S19). Moreover, the upregulated genes in the CC disclosed their activity in the nuclear nucleosome and chromatin. In contrast, the downregulated genes revealed their role in the phagophore and mRNA cleavage and polyadenylation specificity factor complex (Supplementary Document 1 Figure S9 and S18).

### Pathways enrichment analysis

The pathways enrichment analysis showed that the upregulated genes in the cka1Δ condition were associated with glycerophospholipid metabolism, while the downregulated genes were involved in DNA replication and cysteine-methionine metabolism.

The triple condition displayed the involvement of upregulated genes in the glycerophospholipid metabolism, mitophagy, and homologous recombination, whereas downregulated genes were involved in the DNA replication, cysteine and methionine metabolism, and starch and sucrose metabolism.

 In the wild-type strain condition, the upregulated genes pinpointed their major role in the glycosylphosphatidylinositol (GPI)-anchor biosynthesis and DNA replication. In contrast, the downregulated genes were identified to imply interference in nuclear nucleosomes and chromatin. The pathways enrichment is shown in Supplementary Document 1, Figure S21-S23.

### PPI analysis and hub genes identification

Cytohubba, a Cystoscape plug-in, was used to identify hub genes, with the top 10 upregulated genes identified (Q5KKN1_CRYNJ, Q5KA63_CRYNJ, Q5KFT2_CRYNJ, Q5K997_CRYNJ, ARO1_CRYNJ, Q5KD10_CRYNJ, Q5KL19_CRYNJ, Q5KJU6_CRYNJ, Q5KG11_CRYNJ, Q5KC42_CRYNJ) and downregulated hub genes (Q5KP63_CRYNJ, Q5KEV1_CRYNJ, Q5KNH2_CRYNJ, Q5KFT0_CRYNJ, Q5KA46_CRYNJ, Q5KCS1_CRYNJ, Q5KCI8_CRYNJ, Q5KLJ6_CRYNJ, Q5KGV9_CRYNJ, Q5KN65_CRYNJ) were identified in the cka1Δ vs wild-type strain condition.

Moreover, in the triple vs wild-type strain condition, the top 10 upregulated hub genes (Q5K997_CRYNJ, Q5K8G6_CRYNJ, Q5KKN1_CRYNJ, Q5KL19_CRYNJ, Q5K7S9_CRYNJ, Q5KD10_CRYNJ, ARO1_CRYNJ, Q5KC42_CRYNJ, Q5KJ61_CRYNJ, Q5KNI6_CRYNJ), as well as downregulated genes (Q5KJD4_CRYNJ, Q5KCS1_CRYNJ, Q5KK99_CRYNJ, Q5KJU2_CRYNJ, Q5KLJ6_CRYNJ, Q5KNH2_CRYNJ, Q5KMA0_CRYNJ, Q5K979_CRYNJ, Q5K947_CRYNJ, Q5KEV1_CRYNJ), were identified.

Furthermore, the top 10 upregulated hub genes (Q5KP63_CRYNJ, Q5KCS1_CRYNJ, Q5KNH2_CRYNJ, Q5KA46_CRYNJ, Q5KEV1_CRYNJ, Q5KFT0_CRYNJ, Q5KAB9_CRYNJ, Q5KN73_CRYNJ, Q5KLJ6_CRYNJ, Q5KHQ2_CRYNJ) and downregulated genes (Q5K997_CRYNJ, Q5KKN1_CRYNJ, Q5KIM6_CRYNJ, Q5KA63_CRYNJ, ARO1_CRYNJ, Q5KL19_CRYNJ, Q5KD10_CRYNJ, Q5KFT2_CRYNJ, Q5KCT5_CRYNJ, Q5KJ92_CRYNJ) identified for the wild-type strain vs all mutants. The top 10 hub genes are illustrated in Supplementary Document 1 Figure S24(A-C), Tables S1-S6.

### Structural retrieval and virtual screening against natural compounds

The 3D structures of the target proteins, except *cka1*, were retrieved from AlphaFold, while the 3D structure of *cka1* was retrieved from the PDB database (PDB ID: 6K3L). Moreover, the domains of the target proteins were identified from the InterPro database. The proteins, Q5KHQ2_CRYNJ, Q5KN73_CRYNJ, Q5KFT0_CRYNJ, Q5KA46_CRYNJ, ckb1, and ckb2 showed no domains. The details of protein structure retrieval and domain analysis are mentioned in Supplementary Document 2, Table S1-S2, and Figure S1.

**Figure 1:**
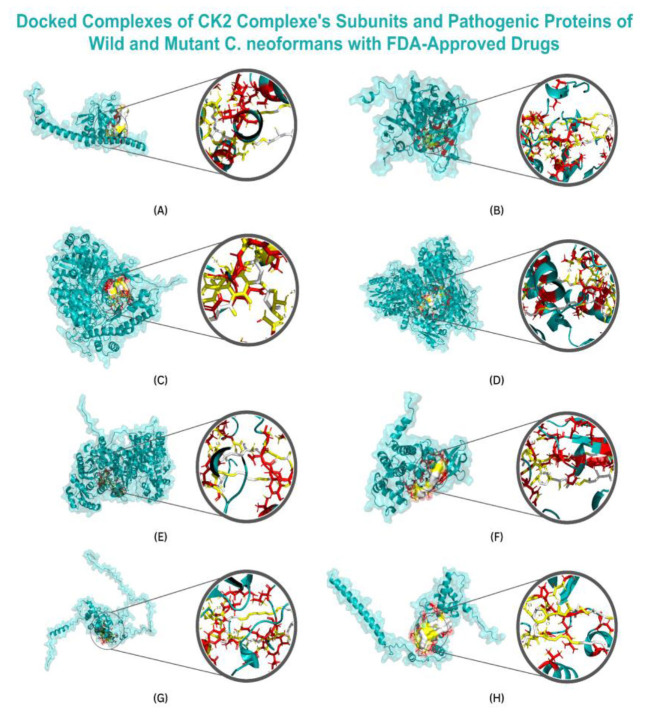
Depicting the surface along with the zoomed view of the target proteins with their top-first compounds.

**Figure 2:**
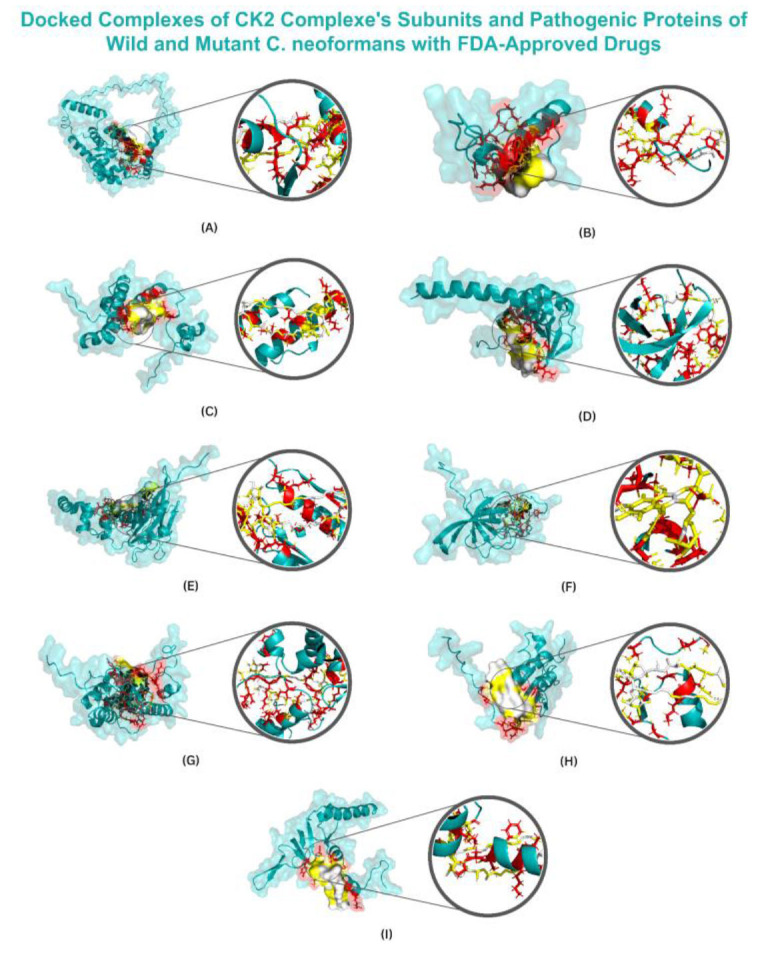
Illustrating the surface along with the zoomed view of the target proteins with their top-first compounds.

Moreover, the ARO1_CRYNJ showed the highest binding affinity of -13.89 kcal/mol against CHEMBL267345, while the cka1, Q5KNI6_CRYNJ, Q5KC42_CRYNJ, Q5KFT2_CRYNJ, ckb1Δ, Q5KCS1_CRYNJ, Q5KEV1_CRYNJ, ckb2Δ, Q5KFT0_CRYNJ, Q5KLJ6_CRYNJ Q5KHQ2_CRYNJ exhibited the highest binding affinity of -13.36, -12.81, -12.65, -12.26, -11.91, -11.49, -11.4, -11.15, -9.87, -9.75, and -9.55 in kcal/mol, respectively with CHEMBL267345. Furthermore, Q5KL19_CRYNJ and Q5KNH2_CRYNJ exhibited -12.76 and -10.89 affinities against CHEMBL1117, while Q5KA46_CRYNJ and Q5KAB9_CRYNJ showed affinities of -10.93 and -10.01 against CHEMBL1200647, respectively, as demonstrated in Supplementary Document 2 Table S3. The docked complexes are shown in Figures 1 and 2.

Additionally, it is computationally and experimentally reported that AMPHOTERICIN B shows stronger binding to ergosterol than cholesterol, suggesting as the basis of its specificity as an antifungal agent (https://www.mdpi.com/2079-4991/10/12/2439). Moreover, IDARUBICIN exhibits a half-maximal inhibitory concentration (IC_50_) value of 2.1-4.7 µM in ATPase and DNA supercoiling assays (https://portlandpress.com/biochemj/article-abstract/477/21/4167/226605/Structure-based-drug-repurposing-to-inhibit-the). Lastly, it was shown in a study that CANDICIDIN showed a binding affinity of -13.84 Kcal/mol with *Tc24* protein (https://www.mdpi.com/2076-393X/11/2/267).

### Protein-ligand interaction analysis

The cka1 exhibited binding within the Protein kinase domain, while Q5KFT2_CRYNJ exhibited binding within the Carbamoyl-phosphate synthetase large subunit-like, ATP-binding domain and the Glyceraldehyde 3-phosphate dehydrogenase, NAD(P) binding domain. Moreover, IDARUBICIN interacted within the Glycine cleavage system P-protein, N-terminal domain and Glycine dehydrogenase, C-terminal domain for Q5KL19_CRYNJ, while within the Small ribosomal subunit protein uS17, N-terminal domain for Q5KNH2_CRYNJ. Additionally, the Q5KA46_CRYNJ exhibited no domain interaction with CANDICIDIN, while Q5KAB9_CRYNJ exhibited binding within the Large ribosomal subunit protein eL14 domain and KOW motif. The interactions of protein and ligand are illustrated in (Supplementary Document 2, Figures S2-S18). The interacting binding residues and their positions for each target protein are mentioned in Supplementary Document 2 Table S4.

## Discussion

 Fungal infectious diseases are the major contributor to deaths across the globe [ 31 ]. Cryptococcal meningitis, a fatal disease caused by *C. neoformans*, is currently untreated, and the current therapies against the infection are ineffective [ 32 ]. The Ck2 complex in *C. neoformans*, consisting of cka1, ckb1, and ckb2, regulates cellular processes like proliferation, signaling pathways, angiogenesis, cell growth, and apoptosis.[ 33 ]. Understanding the Ck2 complex's functional and structural aspects is crucial for targeting pathogenic subunits and genes responsible for fungi virulence, particularly in *C. neoformans*.

This study examined RNA-sequenced cell cultures of C. neoformans under various conditions, revealing dysregulated genes affecting specific pathways. Glycerophospholipid metabolism was the most dysregulated pathway in two conditions, while Glycosylphosphatidylinositol (GPI)-anchor biosynthesis was the major dysregulated pathway in all mutants.

 Studies suggest that *C. neoformans* metabolize lipids to promote pathogenicity in hosts, with extracellular phospholipases potentially causing tissue damage contributing to virulence [ 34 ]. A study found that the functioning of glycerophospholipid pathway metabolites like phosphatidylcholine, phosphatidylethanolamine, phosphoinositide, and phosphatidic acids enabled *S. cerevisiae* to sustain energy production. [ 35 ]. However, the glycosylphosphatidylinositol (GPI)-anchor biosynthesis pathway is critical in signaling, cell growth, cell development, and other processes [ 36 ]. The study suggests that addressing the genes/proteins involved in the survival and pathogenicity of C. neoformans could be a promising method for inhibiting or killing pathogenic C. neoformans.

Moreover, this study identified potential FDA-approved drugs, such as Amphotericin B, an antifungal agent reported to mediate killing in *C. neoformans* through the induction of a strong oxidative burst [ 37 ]. It has a broad-spectrum antifungal effect, with a low incidence of clinical resistance, and is particularly important in clinical practice for treating invasive fungal infections [ 38 ]. Amphotericin B was found to be effective in treating 13 proteins, including pentafunctional AROM polypeptide, CMGC/CK2 protein kinase, 60s ribosomal protein l7 putative, glyceraldehyde-3-phosphate dehydrogenase, pyruvate carboxylase, casein kinase II subunit beta, 60S ribosomal protein L8, ribosomal protein S2, casein kinase II subunit beta, and large ribosomal subunit protein eL39.

Furthermore, Idarubicin is an anthracyclin antileukemic drug that interacts with the DNA topoisomerase II and is a clinically effective quinone-containing anticancer agent used in treating various human cancers [ 39 , 40 ]. Additionally, a study reported a promising antifungal activity of idarubicin against several species of fungi, such as *A. niger*, *C. glabrata*, and *C. neoformans* [ 41 ], Idarubicin may be effective against P protein and S11 putative protein, while Candicidin, an antifungal compound from Streptomyces griseus, has practical applications in treating infectious diseases caused by fungi, particularly C. albicans [ 40 ]. Candicidin may be an effective drug against ribosomal proteins S18 and putative proteins.

Conclusively, Amphotericin B, idarubicin, and candicidin are the top drugs for each protein, with Candicidin showing more potency and susceptibility to selected proteins. Amphotericin B demonstrated the most inhibition potential. Additionally, experimental investigations are necessary for the corroboration of the inhibition of candidate therapeutic target proteins with amphotericin B, idarubicin, and candicidin compounds.

## Conclusion

The Ck2 complex in *C. neoformans* plays a crucial role in its pathogenesis, regulating cell processes like proliferation, signaling pathways, growth, angiogenesis, and apoptosis. Therefore, this computational study reveals the top-performing drugs such as amphotericin B, idarubicin, and candicidin for each protein. These drugs can target potential pathogenic proteins responsible for *C. neoformans'* virulence, and their effects on these proteins can be further confirmed through clinical investigations.
